# *CCL3L1* copy number, HIV load, and immune reconstitution in sub-Saharan Africans

**DOI:** 10.1186/1471-2334-13-536

**Published:** 2013-11-12

**Authors:** Eleni Aklillu, Linda Odenthal-Hesse, Jennifer Bowdrey, Abiy Habtewold, Eliford Ngaimisi, Getnet Yimer, Wondwossen Amogne, Sabina Mugusi, Omary Minzi, Eyasu Makonnen, Mohammed Janabi, Ferdinand Mugusi, Getachew Aderaye, Robert Hardwick, Beiyuan Fu, Maria Viskaduraki, Fengtang Yang, Edward J Hollox

**Affiliations:** 1 Department of Clinical Pharmacology, Karolinska Institutet, Stockholm, Sweden; 2 Department of Genetics, University of Leicester, University Road, Leicester, LE1 7RH, UK; 3 Department of Pharmacology, Addis Ababa University, Addis Ababa, Ethiopia; 4 Unit of Pharmacology, School of Pharmacy, Muhimbili University of Health and Allied Sciences, Dar es Salaam, Tanzania; 5 Internal Medicine, Addis Ababa University, Addis Ababa, Ethiopia; 6 Institution of Medicine, Unit of Infectious Diseases, Karolinska Institutet, Karolinska University Hospital, Huddinge, Sweden; 7 Department of Internal Medicine, Muhimbili National Hospital, Dar es Salaam, Tanzania; 8 Department of Internal Medicine, Muhimbili University of Health and Allied Sciences, Dar es Salaam, Tanzania; 9 Wellcome Trust Sanger Institute, Hinxton, Cambridge, UK; 10 College of Medicine, Biological Sciences and Psychology, University of Leicester, Leicester, UK

## Abstract

**Background:**

The role of copy number variation of the *CCL3L1* gene, encoding MIP1α, in contributing to the host variation in susceptibility and response to HIV infection is controversial. Here we analyse a sub-Saharan African cohort from Tanzania and Ethiopia, two countries with a high prevalence of HIV-1 and a high co-morbidity of HIV with tuberculosis.

**Methods:**

We use a form of quantitative PCR called the paralogue ratio test to determine *CCL3L1* gene copy number in 1134 individuals and validate our copy number typing using array comparative genomic hybridisation and fiber-FISH.

**Results:**

We find no significant association of *CCL3L1* gene copy number with HIV load in antiretroviral-naïve patients prior to initiation of combination highly active anti-retroviral therapy. However, we find a significant association of low *CCL3L1* gene copy number with improved immune reconstitution following initiation of highly active anti-retroviral therapy (p = 0.012), replicating a previous study.

**Conclusions:**

Our work supports a role for *CCL3L1* copy number in immune reconstitution following antiretroviral therapy in HIV, and suggests that the MIP1α -CCR5 axis might be targeted to aid immune reconstitution.

## Background

AIDS, caused by the retrovirus HIV, is predicted by 2030 to become globally the single largest cause of morbidity, as measured by disability-adjusted life-years [1]. African countries currently have the highest disease burden of HIV, with 9.2% prevalence in Addis Ababa in Ethiopia and over 10% in Dar-es-Salaam in Tanzania, yet almost all genetic studies have focused on cohorts from Western countries [2]. The genetic architecture of HIV susceptibility in Africans is likely to be different to Europeans, yet genome-wide association studies of host susceptibility to HIV have not yielded any significant results [3]. These studies miss regions that show copy number variation, particularly structurally complex regions that are not correlated with alleles at flanking SNP markers [4].

Copy number variation (CNV) is defined as the variation in copy number of a given DNA sequence in a diploid genome. CNV is common in the genome, affects gene expression, and involves immune response genes [5-7], suggesting that it may affect susceptibility of the host to infectious disease. CNV of the killer cell immunoglobulin receptor genes has been shown to affect host control of HIV infection, as determined by the viral load (VL) at setpoint [8], and we have recently shown association of β-defensin CNV both with HIV viral load at initiation of highly-active anti-retroviral therapy (HAART) and with consequent immune reconstitution [9].

The genes *CCL3L1*/*CCL4L1* encode the chemokines MIP-1α and MIP-1β which are both ligands for the chemokine receptor CCR5 used as a co-receptor by R5 strains of HIV. These genes show CNV, and this has been shown to affect HIV acquisition, progression to AIDS, and immune reconstitution following highly active anti-retroviral therapy (HAART) [10-12]. An attractive model is that these chemokines and HIV compete for the same receptor CCR5, and that increasing copy number increases the levels of chemokine, thereby increasing competition with HIV for the receptor [13]. A gene dosage effect linking gene copy number and protein levels is needed to support this hypothesis, and evidence has been contradictory. Early studies supported a gene dosage effect [10,11], but recent studies have suggested that the influence of extra gene copies on total protein levels is low [14,15]. A problem in these experiments is that the protein product of *CCL3* (called MIP1α-LD78α) and *CCL3L1* (MIP1α-LD78β) cannot be discriminated using standard antibodies. Thus analyses using antibody-based detection of protein products may not detect a gene dosage effect, particularly given the higher levels of *CCL3* transcription and presumably MIP1α-LD78α in the blood. Although both protein isoforms signal through CCR5, only the LD78β isoform can be cleaved by dipetidyl peptidase IV to generate a monocyte attractant and CCR1 agonist [16,17]. Indeed, functional evidence remains supportive: measuring the chemotactic response of cells to supernatants from lipopolysaccharide-stimulated monocytes from different individuals supports an effect of different *CCL3L1* gene copy number [10]. However, other mechanisms for an effect of *CCL3L1* copy number can be invisaged, either directly or indirectly by affecting other immunological phenotypes such as the CD4+ cell count.

Attempts at replicating the genetic association of *CCL3L1* copy number and HIV susceptibility have yielded contrasting results. A meta-analysis of nine studies has supported an association of lower *CCL3L1* with susceptibility to HIV [18], but this study did not critically analyse the quality of the published data used in the meta-analysis. For example, the use of quantitative PCR to determine *CCL3L1* copy number may generate false-positive associations [19-21]. It may be that *CCL3L1* and *CCL4L1* do not always vary in copy number as a block, which might explain at least some of the heterogeneity in results when different methods are used to determine copy number. However, when more robust reliable methods are applied to large European cohorts there is no evidence of this, suggesting that when measured with sufficient precision and accuracy, *CCL3L1* and *CCL4L1* covary as a block [22,23]. In common with most of the literature, we refer to this copy number variation as *CCL3L1* copy number variation, but it should be remembered that it also involves *CCL4L1* and possibly *TBC1D3*.

*CCL3L1* CNV has also been associated with a variety of other infectious diseases, including tuberculosis [24], hepatitis B [25], hepatitis C [26] and Kawasaki Disease [27]. Such association studies are almost always small, use qPCR to type copy number, not necessarily replicated [28], and in some cases the reported association is seen only on a background of a particular genotype at another locus. While such studies are based on reasonable hypotheses concerning the function and interaction of proteins and pathogens, the marginal significance levels and limited power of such studies means that drawing definitive conclusions regarding the role of genetic variation remains difficult. In the most technically- and genetically-thorough study to date, a weak suggestive association with protection from anemia in malarial infection was found, but this family-based study too lacked power to detect anything but strong effects [29].

Evidence from other African studies of *CCL3L1* and HIV has been contradictory. In a small Zimbabwean longitudinal cohort, no association of *CCL3L1* copy number with HIV status or progression was found [30]. However, analysis of mother-to-child transmission in South Africa suggested that higher copy number was protective against HIV transmission [31]. In this context, we decided to analyse our previously described cohort of HIV patients from Ethiopia and Tanzania for association of *CCL3L1* copy number with viral load immediately prior to HAART and immune reconstitution during HAART. African populations are known to have a higher average copy number than European populations [11,31], due either to natural selection or genetic drift. This has the advantage, in an association study context, of providing a wider range of copy number and therefore a potentially larger gene dosage effect. However, there are significant technical challenges in accurately typing multiallelic copy numbers at this, or indeed other, loci. We decided to use the paralogue ratio test (PRT) to determine copy number, which is the most robust technique available for typing this locus on large cohorts [19,21].

## Methods

### Sample collection

Patient sample, DNA extraction and clinical data collection was as previously described [9,32,33]. The study protocol was approved by the Institutional Review Board at the Faculty of Medicine, Addis Ababa University and Ethiopian Science and Technology Ministry; the regional ethical review board in Stockholm at the Karolinska Institutet and the ethical review committee of Muhimbili University of Health and Allied Sciences. Written informed consent was obtained from each subject before the start of this study. DNA samples from the HapMap YRI population (Yoruba from Ibadan, Nigeria) were obtained from Coriell Cell Repositories (Camden, NJ, USA).

The CCR5 δ32 allele, associated with protection from HIV infection and disease, progression, was not detected in either the Tanzanian or Ethiopian samples [9], where the deletion allele is known to be protective against HIV progression. Patient numbers used at each stage of the study are given in Additional file 1: Table S1. Baseline characteristics of patients are given in Additional file 2: Table S2.

### Copy number typing

Copy number typing was performed using the PRT approach described previously [22]. Briefly, data from three separate PRT assays measuring copy number across the *CCL3L1* segmental duplication are normalised using four known positive control samples (C0075 – 1 copy, C0150 – 2 copies, C0007 – 3 copies, C0877 – 4 copies), available as part of the human reference control plate HRC-1 from the Health Protection Agency, Porton, UK, analysed with every experimental PCR plate, and then averaged to give an unrounded estimate of copy number. The replicate testing of the four positive controls using the three separate PRT assays produces datapoints that clearly cluster, with clusters showing a linear relationship with copy number, and no assay-specific biases in clustering (Additional file 3: Figure S1).

Samples were tested in duplicate if the coefficient of variation of the values from the three separate assays exceeded a given threshold, typically 0.2, and the result that gave the lower coefficient of variation taken on to the next stage of analysis. A small number of samples gave consistently high (>0.5) coefficient of variation scores even after repeated testing. This was due to altered copy number of either LTR16 or CCL4, and for these the copy number from the two consensus PRTs was taken forward. However, in general, raw copy number estimates from each of the three PRT assays was highly concordant across samples, with clustering about integer copy numbers evident at lower copy numbers (Additional file: 4 Figure S2).

Integer copy numbers were inferred from mean unrounded copy number estimates using a Gaussian mixture model, implemented in the statistical language R (package CNVtools [34]). The appropriateness of using Gaussian distributions to model PRT data can be tested by analysis of the data from the positive control samples analysed on every PCR plate. When normalised by copy number, it is clear that the combined dataset fit the Gaussian distribution well, although a number of outliers are seen (Additional file 5: Figure S3a). These outliers seem to be more likely for the lower copy number samples, suggesting that the assumption of the Gaussian distribution not only holds but may even be a stronger assumption for PRTs measuring higher copy numbers (Additional file 5: Figure S3b). A mixture model of nine components was fitted, based on observation of the data and prior studies. The model of variance components was fixed to have similar variance, an assumption supported by the similar variance of the repeated PRT values from the four positive controls. The resulting clustering quality score (Q) was 3.9. A posterior probability of the integer copy number call being correct was given for each sample. Where this probability was below 0.8, and the probability of the copy number one higher or one lower was therefore >0.2, then the mean of a duplicate test (if carried out) was used to call the correct integer copy number.

### Fibre FISH

Fibre-FISH was performed as described previously [35]. Briefly, stretched DNA fibers were prepared from lymphoblastoid cell lines. Fosmid DNA was prepared using the Phase-Prep BAC DNA kit (Sigma-Aldrich) following the manufacturer’s protocol. Fosmids used were G248P85689G4 (white, hg18 chr17:31434865–31475400), G248P84883A8 (green, hg18 chr17:31468941–31505286) and G248P8961D8 (red, maps to hg18 twice at chr17:31537181–31574736 and chr17:31638770–31676303). The green clone was labelled with Dinitrophenol (DNP)-11-dUTP (PerkinElmer) and detected with rabbit anti-DNP and Alexa 488 conjugated goat anti-rabbit IgG. The red clone was labelled with Digoxigenin (DIG)-11-dUTP (Roche) and detected with monoclonal mouse anti-DIG IgG (Sigma-Aldrich) and Texas red conjugated donkey anti-mouse IgG (Invitrogen). The white clone was labelled with biotin-16-dUTP and detected with one layer Cy3-avidin. After detection, slides were mounted with SlowFade Gold® (Invitrogen) mounting solution containing 4′, 6-diamidino-2-phenylindole (Invitrogen). Images were captured on a Zeiss Axioplan fluorescent microscope and processed with the SmartCapture software (Digital Scientific UK).

### Statistical analysis

To analyse the effect of *CCL3L1* on HIV load at initiation of HAART, we initially constructed a generalised linear model using SPSS 20.0 (IBM) and a gamma-identity link, as previously published. This link function did not model the data (which included new clinical data) well, and a gamma-log link provided a better fit to the data. Notably, for the previously-published β-defensin dataset [9], both gamma-identity and gamma-log links model the data well, and although the gamma-identity model was chosen, the two models are almost indistinguishable based on several goodness-of-fit criteria and report very similar significance levels, therefore not calling our previous results into question. The model was calculated using type III sum of squares ANOVA, with goodness-of-fit analysed using Wald statistics.To examine the effect of *CCL3L1* copy number on CD4+ count following initiation of HAART, we constructed a generalised linear mixed model, using STATA, where the dependent variable (CD4+ count) was modelled as a Gaussian distribution. In this model, we assigned population and disease status as fixed factors, initial CD4+ count and time since HAART initiation as scalar covariates and integer copy number as an ordinal covariate. The model was calculated using type III sum of squares ANOVA, with a variance correction to allow for multiple CD4+ timepoint readings from a single patient.

## Results

### Analysis and validation of copy number typing

**Figure 1:**
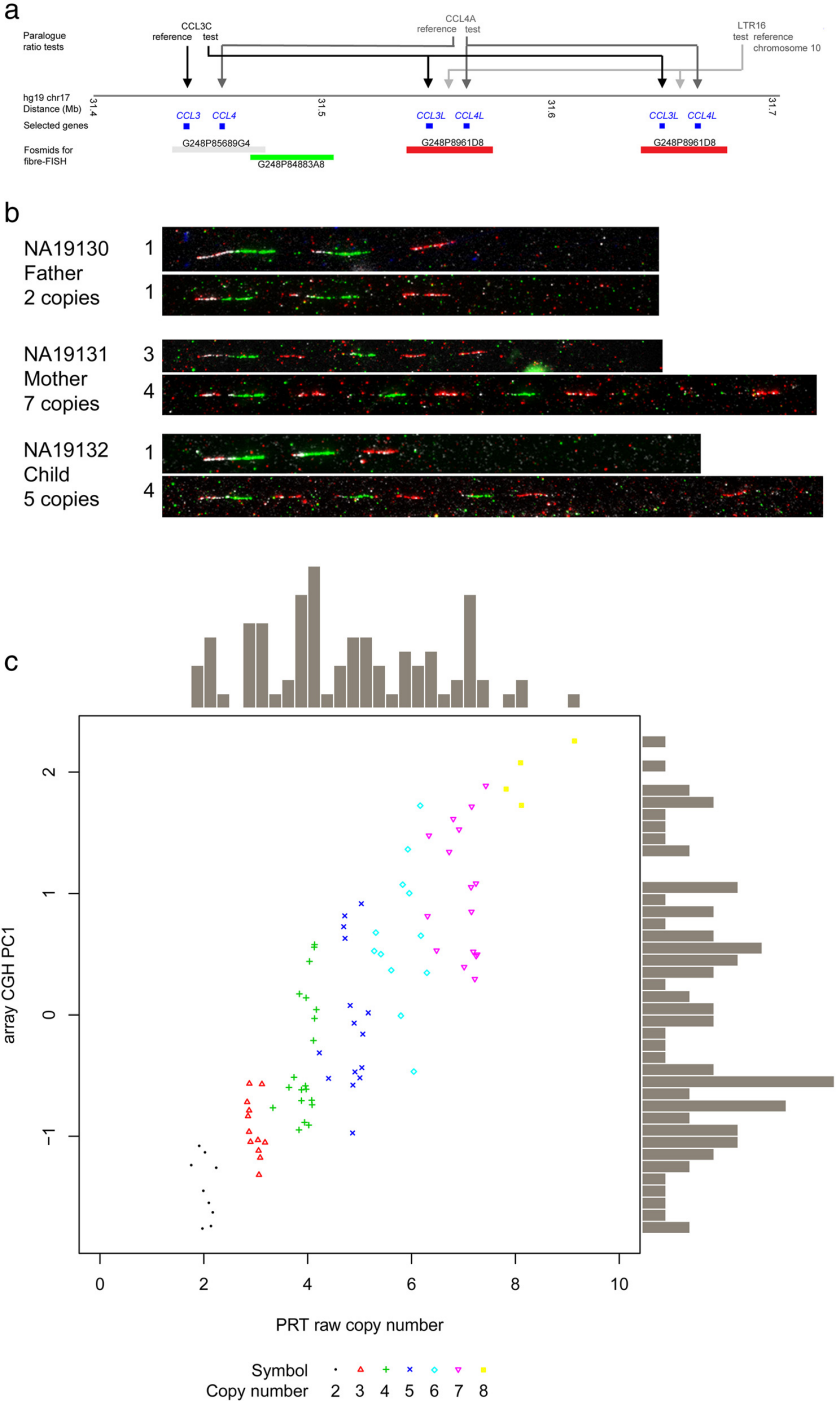
**Validation of CNV using array CGH and fibre-FISH. a)** The reference genomic region studied, showing the location of the sequences used in the paralogue ratio test (PRT) and the fosmids used for fibre-FISH analysis. **b)** Fibre-FISH analysis on stretched DNA fibres from three lymphoblastoid cell lines using the fosmid probes shown in part a). The three cell lines are from a YRI parent–child trio recruited for the HapMap project, with their DNA sample IDs given. PRT copy number estimates are given under each ID, and copy number estimated from each stretched individual chromosome given immediately to the left of a representative fiber-FISH image. **c)** Comparison of raw PRT estimates of *CCL3L1* copy number on HapMap YRI samples (x-axis) with estimates from arrayCGH data (y-axis). Points are coloured according to final integer copy number estimates, as indicated by the key below the scatterplot. PC1=first principal component of arrayCGH data.

We used a previously-published and well-established method for copy number typing, called the paralogue ratio test (PRT) to type *CCL3L1* copy number (Figure 1a).

**Figure 2:**
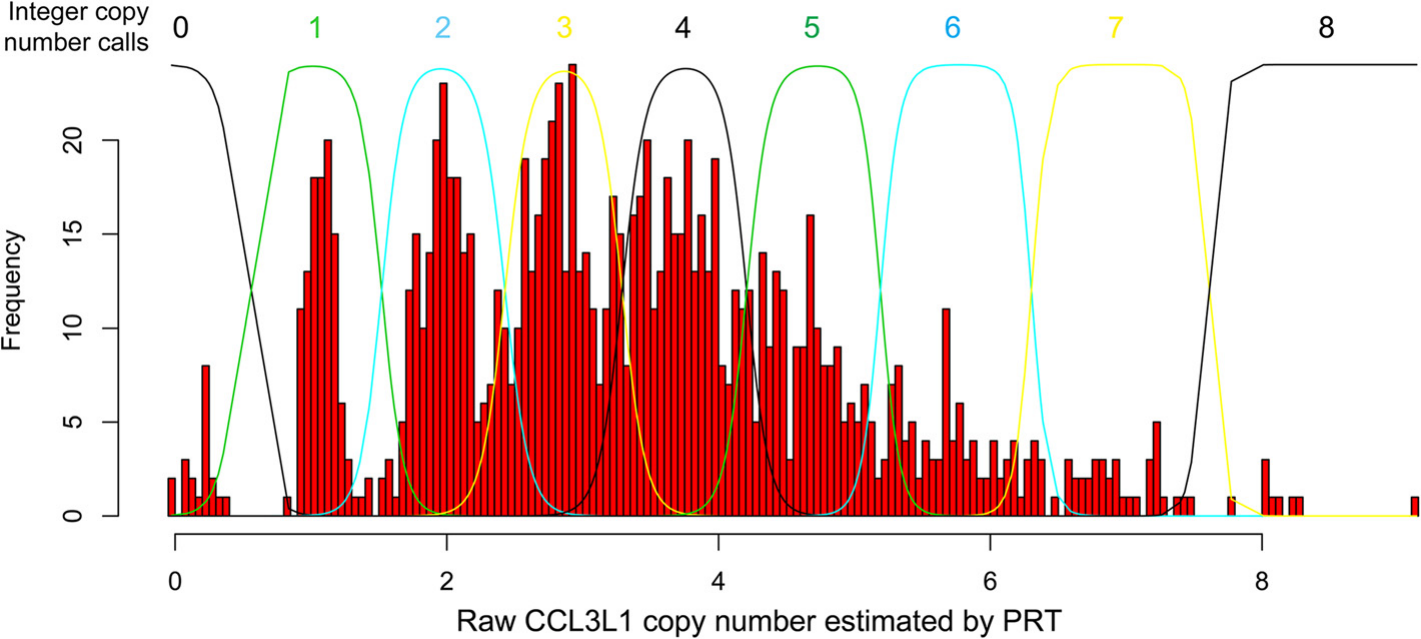
**Calling integer *CCL3L1 *copy number from raw PRT data.** The histogram shows the copy number distribution of 1133 individuals. A Gaussian mixture model, with seven components, is fitted to the data, and each individual component is plotted with the corresponding integer copy number shown above each peak.

1134 samples were tested in total, and integer copy number called using a Gaussian mixture model approach (Figure 2, see Methods) after removal of one outlier with very high copy number (~14). 192 (16.9%) samples gave an integer copy number call posterior probability of less than 0.8. Of these, 57 (30%) had been tested in duplicate, of these 57 duplicates, 34 (60%) supported the original copy number call and 23 supported the alternative copy number call (Additional 6: Figure S4). If we conservatively assume that there is no correlation between the samples selected for duplicate testing because of high coefficient of variation values and the samples giving posterior probability values <0.8, we can estimate the error rate to be 6.7%, and these errors will involve an incorrect call of +/- 1 copy number. Error rate is likely to be significantly lower than this because samples were selected for duplicate testing based on high coefficient of variation values, and are therefore likely to be significantly enriched for miscalled samples.

To validate our copy number calling at higher copy numbers, we used fibre-FISH on extended DNA fibres from cell lines derived from a parent–child trio from the YRI HapMap population. Our estimates, estimated from PRT prior to fibre-FISH analysis, agreed with the number of copies determined by FISH (Figure 1b). This is shown by the fosmid probe labelled red, which maps to the *CCL3L1* repeat and has been used previously to estimate copy number in humans [35]. Interestingly, of the 14 *CCL3L1* repeats directly visualised in these three trios, 11 have a repeat structure that includes a fosmid probe, labelled in green, which covers a region between the CCL4 gene and the *TBC1D3* and *CCL3L1* genes. This is in contrast to *CCL3L1* repeats previously visualised in Europeans, where the repeat appears to be represented just by the red-labelled probe, and has been estimated to be 90 kb in size. Therefore, in Yoruba at least, and perhaps in other sub-Saharan Africans, there appears to be heterogeneity in *CCL3L1* repeat structure not yet observed in Europeans.

Concordance of the three PRT assays is an important test of heterogeneity of the repeat. Studies of *CCL3L1*/*CCL4L1* copy number in European populations using the PRT method by us (unpublished data) and others [23] have shown concordance between all three probes, supporting the idea that this region is copy number variable en bloc and averaging the values of the three probes accurately reflects the copy number of this block. However, in this study, a small number of Ethiopian samples gave consistently high coefficient of variation scores due to one probe repeatedly giving discordant results. These can be seen as outliers on Additional file 4: Figure S2, and example data from samples are given in Additional file 7: Figure S5. This suggests either sub-Saharan African-specific copy number heterogeneity or rare duplication of the PRT reference locus, and is likely to contribute to the error rate observed in these data. All three PRT assays map to the region represented by the red fosmid probe in fibre-FISH, and this PRT heterogeneity was not observed in the YRI or Tanzanian samples, strongly suggesting that this heterogeneity is of a different nature to that observed by fiber-FISH, and is perhaps confined to the Ethiopian population.

To further validate our *CCL3L1* copy number calls, for the YRI HapMap samples we compared our estimates with arrayCGH data previously generated using the Agilent 210 K CNV chip [6]. There is a clear positive correlation between the two methods, and it is also clear that the PRT generates data that clusters effectively into integer copy numbers, particularly at lower copy numbers, in contrast to aCGH where there is considerable overlap of copy number classes (Figure 1c).

### CCL3L1 copy number distribution in different populations

The copy number distributions are shown in Table 1. As has been observed previously, the copy number range for all three African populations is higher than European populations, where the common copy number range is between 1 and 4 copies per diploid genome [22]. Of the three African populations, the YRI show the highest mean copy number, although the Ethiopian population shows the greatest range (between 0–8 copies) and one Tanzanian shows a particularly high copy number of 14.

**Table 1:** ***CCL3L1 *** **Copy number distribution and comparisons between populations**

***CCL3L1 *** **copy number**	**Tanzanian HIV**		**Tanzanian HIV + TB**		**Ethiopian HIV**		**Ethiopian HIV + TB**		**YRI unrelated**	
0	0	0	0	0	7	0.03	11	0.03	0	0
1	2	0.01	2	0.01	40	0.17	46	0.14	7	0.12
2	25	0.12	13	0.09	39	0.17	71	0.22	8	0.14
3	52	0.25	40	0.28	41	0.17	86	0.26	13	0.22
4	56	0.27	48	0.33	42	0.18	55	0.17	10	0.17
5	43	0.21	22	0.15	33	0.14	35	0.11	9	0.16
6	22	0.11	15	0.10	21	0.09	17	0.05	8	0.14
7	4	0.02	5	0.03	8	0.03	5	0.02	3	0.05
8	1	0	0	0	4	0.02	0	0	0	0
9	0	0	0	0	0	0	0	0	0	0
10	0	0	0	0	0	0	0	0	0	0
14	1	0	0	0	0	0	0	0	0	0
N	206		145		235		326		58	
mean	4.024		3.966		3.353		3.000		4.72	

There is a small but marginally significant difference (p = 0.02, t-test) between the mean copy number of the Ethiopian HIV-only and the HIV-TB co-infected cohort, although this is not replicated in the smaller Tanzanian cohort (p = 0.69). Indeed analysis by combining CNV calling and association testing using CNVtools, which can account for differential bias effects between cohorts, reported a non-significant effect for the Ethiopian dataset (p = 0.52), suggesting a very subtle technical bias between the DNA plates containing HIV-only samples and those containing HIV-TB samples.

### Association of copy number with clinical parameters

To investigate the effect of *CCL3L1* copy number on viral load, immediately prior to HAART, we fitted a generalised linear model to the data, with population of origin, tuberculoisis co-infection status and CD4+ count immediately prior to HAART as cofactors. We found significant association with population of origin, TB infection and CD4+ count, but no effect of *CCL3L1* copy number (Table 2). We repeated the analysis using raw copy number values, with no change.

**Table 2:** Model fitting – output viral load

**Model**	**β coefficient (95% CI) (copies/mL)**	**P value**
Population	−0.67 (−0.97, -0.36)	<0.001
No TB Co-infection	−0.42 (−0.69, -0.16)	0.002
CD4+ count (cells/mm^3^)	−0.003 (−0.006, -0.001)	0.008
*CCL3L1* copy number	−0.068 (−1.45, 0.009)	0.084

(n = 656 observations).

**Figure 3:**
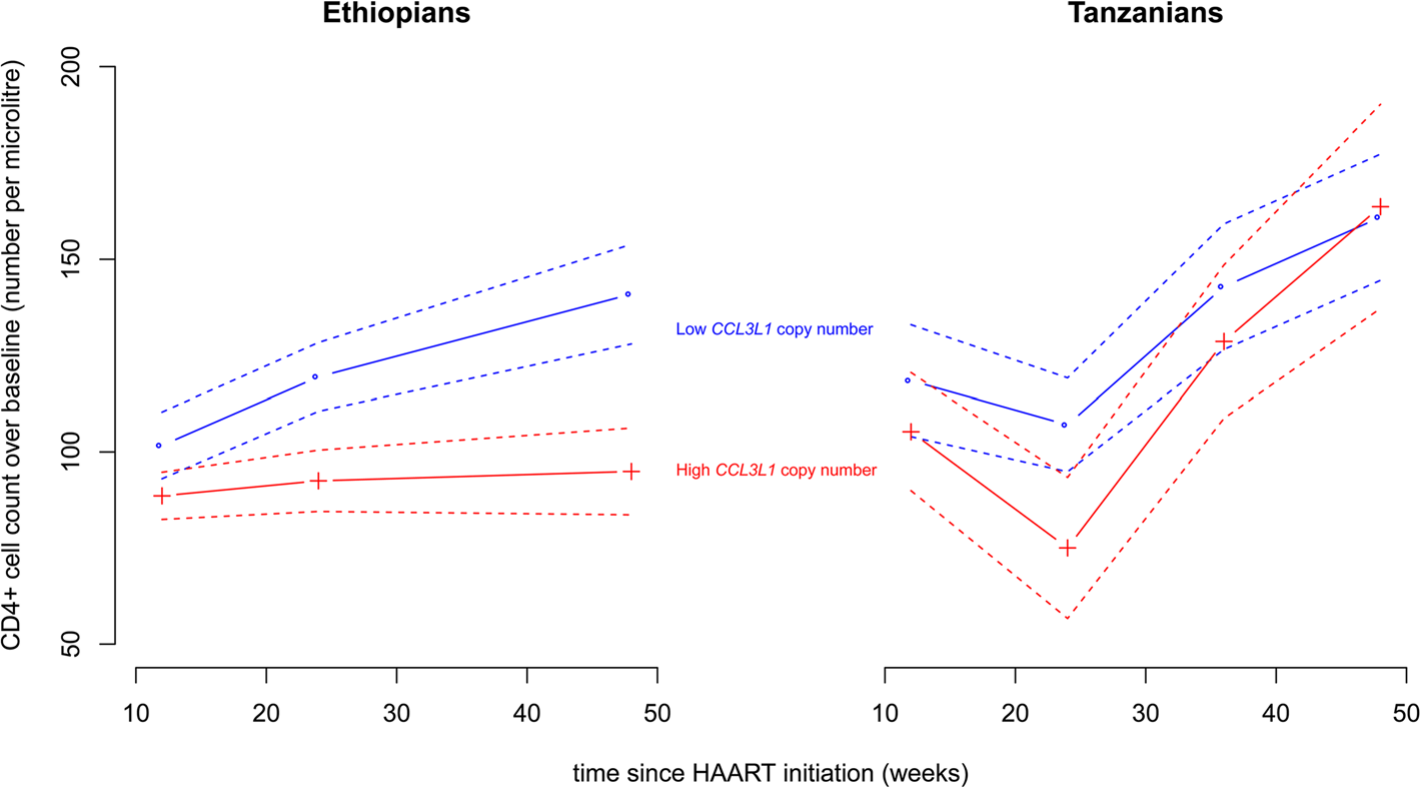
**Response to HAART in Ethiopians and Tanzanians stratified by *CCL3L1 *copy number.** Average values (solid line) and standard error of the mean (dashed line) of CD4+ cell counts was calculated for the different timepoints following initiation of HAART. Patients were stratified according to *CCL3L1* copy number, with high copy number being greater than the median integer value, which was 3 copies in Ethiopians and 4 copies in Tanzanians. N = 798 CD4+ values in Ethiopians, n = 894 CD4+ values in Tanzanians.

To investigate the effect of *CCL3L1* copy number on immune reconstitution following HAART, we measured CD4+ count at 12, 24, 36 and 48 week intervals following initiation of treatment. Using a multivariate linear mixed effects model to control for the multiple repeated measurements contributed by the same patient at different timepoints, we found a significant association of time since initiation of treatment, CD4+ levels at initiation of treatment, population of origin, TB co-infection status and *CCL3L1* copy number (p = 0.012, Table 3, Figure 3). The direction of effect of *CCL3L1* copy number, with higher copy number associated with poorer immune reconstitution, agrees with previous studies [12].

**Table 3:** Model fitting – output CD4 count after HAART

**Model**	**β coefficient (95% CI) (cells/mm**^**3**^**)**	**P value**
Time after HAART (weeks)	2.61 (2.37,2.85)	<0.001
Baseline CD4+ (cells/mm^3^)	0.88 (0.77,1.00)	<0.001
Population	19.36 (5.08,33.63)	0.008
No TB co-infection	16.64 (3.33,29.95)	0.014
*CCL3L1* copy number	−4.75 (−8.46, -1.05)	0.012

N = 1692 observations on 491 patients, mixed effects model accounting for repeat measures at different timepoints.

## Discussion

It has been observed previously that, despite HAART being effective at reducing HIV load to below measurable levels, CD4+ cell count does not always return to healthy levels [36]. This might be due to a variety of factors, including host genetics and co-infection status. Indeed, we demonstrate in this study (Table 3) that both initial baseline CD4+ cell count and absence of TB have a positive effect on the CD4+ count following initiation of HAART, a commonly used measure of immune reconstitution. The role of host genetic variation in influencing different rates of immune reconstitution during HAART is not well understood, yet is of increasing importance as HAART programmes are initiated and continued in areas of high HIV prevalence. Several candidate genes have been suggested to play a role, including a haplotype of the *TRAIL* gene and copy number variation of the β-defensin genes [9,37]. This study suggests that *CCL3L1* copy number has a stronger effect on immune reconstitution than β-defensins (β-defensin β = −3.63 CD4 + cells/ml per copy, *CCL3L1* β = −4.75 CD4+ cells/ml per copy). However, unlike β-defensin copy number, we find no effect of *CCL3L1* copy number on viral load during acute HIV infection, just prior to initiation of HAART.

Previous studies have used combined data from different ethnic groups, with very different *CCL3L1* copy numbers, with HAART started at different CD4 count thresholds. It might be argued that variation in ethnicity was a confounding factor, so that ethnicity rather than *CCL3L1* copy number per se, was responsible for the variation in immunological reconstitution. While in no way a genetically homogeneous cohort, a fact that we attempt to account for in part by using country of origin as a cofactor in our analyses, our study does not combine two dichotomous ethnic groups with very different *CCL3L1* copy number counts and different levels of access to healthcare [12]. Our entire cohort is also completely naïve to antiretroviral therapy prior to initiation of HAART, unlike those previously studied [12,38].

Although we have taken care to ensure the optimum quality of our copy number typing, problems remain particularly in distinguishing higher copy numbers, which are frequent in sub-Saharan African populations. Part of this is technical, due to inherent noise in the assays used, and part biological, due to the variation in repeat structure apparent in certain populations. Both issues cannot be resolved easily without more extensive work on the nature and extent of structural variation at this locus in different populations, and we suggest that this should be a prerequisite before a comprehensive analysis of the clinical role of *CCL3L1* copy number can be made. The Genome Reference Consortium has assembled a reference allele from sequencing BACs from a genomic library derived from a hydatidaform mole, which contains one copy of the *CCL3L1* and *CCL4L1* genes and is likely to represent the most common allele in Europeans (accession number GL383560.1). However we show here that the high-copy alleles characteristic of African populations are not necessarily simply related to the European alleles, and there is clearly a need for accessible physical remapping approaches that can be applied to a significant number of samples to fully characterise structural variation at this locus.

There are three other caveats in interpretation of our study. Firstly, although we control for co-infection with tuberculosis, which represents the major co-morbidity in these populations, we cannot rule out that the effect of *CCL3L1* copy number is indirect, via another infection, rather directly on immune reconstitution. Secondly, as stated previously, the copy number variation involves the genes for the chemokine *CCL4L1*, and *TBC1D3*, a protein involved in macropinocytosis [39]. Although *CCL3L1* is the favoured candidate for mediating the effect of copy number based on the known functional role of the chemokine, a role for the other gene products should not be completely ruled out. Thirdly, we also cannot rule out an indirect effect of *CCL3L1* copy number mediated by an effect on CD4+ levels immediately after seroconversion, which have been shown to affect immune reconstitution [40].

## Conclusions

Taken together, our data support a role for *CCL3L1* copy number in the immune reconstitution following initiation of HAART to treat HIV infection. These data also support the suggestion that treatment of HIV using MIP1α analogues as part of a combined HIV treatment regimen, might adversely affect immune reconstitution, but a small molecule that interferes with MIP1α interactions with cognate receptors might aid immune reconstitution.

## Competing interests

EJH has received grant funding from Pfizer Inc, which had no influence in the conception, design or analysis of this work, and no role in manuscript preparation or publication.

## Authors’ contributions

EA and EJH concieved and designed the study. Experiments were performed by LOH, JB, RH, BF and FY. Data were analysed by EJH, LOH, JB, BF, FY and MV. Clinical data and patient samples were provided by EA, AH, EN, GY, WA, SM, OM, EM, MJ, FM and GA. All authors read and approved the final manuscript.

## Pre-publication history

The pre-publication history for this paper can be accessed here:

http://www.biomedcentral.com/1471-2334/13/536/prepub

## Supplementary Material

Additional file 1: Table S1 Sample sizes used in the study. Arm 3 was recruited with CD4 > 200 and TB, had *CCL3L1* copy number for 96 patients called but was not matched to clinical data for this study. Click here for file

Additional file 2: Table S2 Baseline characteristics of patients analysed. Click here for file

Additional file 3: Figure S1 Analysis of PRT measurement noise in control samples. Individual unrounded PRT values are plotted on the y-axis, according to the different copy numbers of the four controls (x-axis). Each point is coloured according which of the three different PRT assays generated it, all three assays measuring *CCL3L1* copy number. Click here for file

Additional file 4: Figure S2 Clustering of PRT raw data between different assays. For the complete dataset (n = 1133), density scatterplots were draw comparing each of the three different assays with each other. Axis labels indicate raw PRT values, and the colour bar on the left indicates the density of individual datapoints. One extreme point has been omitted. Click here for file

Additional file 5: Figure S3 Analysis of the distribution of PRT values about a single copy number. **a)**. The density of raw unrounded PRT values of the control samples, shown in supplementary Figure 1, is plotted, with values normalised to centre on a mean of zero. The red dotted line represents a Gaussian distribution with a mean and standard deviation taken from the PRT data. The blue dashed line represents a Gaussian distribution fitted to the PRT data. **b)**. Gaussian quantile-quantile plot of raw unrounded PRT values of the control samples. Each value is plotted according the copy number of the control sample, as shown in the legend. The straight line is plotted through the first and third quantiles. Click here for file

Additional file 6: Figure S4 Confidence of integer copy number calls from raw PRT data.Raw PRT calls of the entire dataset (average of three PRT assays) are plotted on the x-axis with posterior probability of the resulting integer copy number call on the y-axis. Points plotted as red triangles are those where P < 0.8 with a repeat measurement which gave a different estimate of integer copy number (±1). Points plotted as green crosses are those where P < 0.8 with a repeat measurement which gave the same estimate of integer copy number. Click here for file

Additional file 7: Figure S5 Examples of assay heterogeneity. Six Ethiopian samples are highlighted, together with the raw PRT ratios, coloured by PRT assay, after several repeat tests. Click here for file
