# Supplementary material for: CCL3L1 copy number, HIV load, and immune reconstitution in sub-Saharan Africans
Source: BMC Infect Dis. 2013 Nov 12;13:536. doi: 10.1186/1471-2334-13-536 (PMC3829100; doi:10.1186/1471-2334-13-536)
Supplement: Additional file 1: Table S1 — Sample sizes used in the study. Arm 3 was recruited with CD4 > 200 and TB, had CCL3L1 copy number for 96 patients called but was not matched to clinical data for this study. [file 1471-2334-13-536-S1.doc]

**Additional file 1: Table S1**Sample sizes used in the study

|  |  |
| --- | --- |
| Total number of individuals analysed, excluding duplicates | 1134 |
| Gaussian mixture model calling (included/excluded) | 1133/1 |
| HapMap YRI trio samples | 88 |
| Samples with no matched record | 2 |
| Tanzanian samples (total/ with matched clinical data) | 353/351 |
| Ethiopian samples (total/with matched clinical data*) | 691/561* |
| CD4<200 with baseline VL data (both populations) | 656 |
| CD4<200 with at least 1 CD4 follow-up datapoint (both populations) | 491 |

Arm 3 was recruited with CD4>200 and TB, had *CCL3L1* copy number for 96 patients called but was not matched to clinical data for this study.
