# Supplementary material for: CCL3L1 copy number, HIV load, and immune reconstitution in sub-Saharan Africans
Source: BMC Infect Dis. 2013 Nov 12;13:536. doi: 10.1186/1471-2334-13-536 (PMC3829100; doi:10.1186/1471-2334-13-536)
Supplement: Additional file 2: Table S2 — Baseline characteristics of patients analysed. [file 1471-2334-13-536-S2.doc]

**Additional file 2: Table S**2 Baseline characteristics of patients analysed

|  |  | Ethiopian | Tanzanian |
| --- | --- | --- | --- |
| All samples with matched clinical data | n | 561 | 351 |
|  | CD4 cells/mm^3^ (mean, sd) | 95.2, 54.2 | 102, 65.1 |
|  | VL copies/mL (x10^5^ , mean, sd) | 3.07, 6.07 | 5.88, 17.8 |
|  | Sex (%male) | 40% | 41% |
|  | Age (mean,sd) | 35.8,9.6 | 39.0, 10.0 |
| CD4<200 with baseline VL | n | 483 | 173 |
|  | CD4 cells/mm^3^ (mean, sd) | 93.8,52.5 | 93.0, 61.3 |
|  | VL copies/mL (x10^5^ , mean, sd) | 3.10, 6.12 | 5.96, 18.1 |
|  | Sex (%male) | 39% | 43% |
|  | Age (mean,sd) | 35.9, 9.4 | 38.9, 9.5 |
