# Supplementary figures and images for: CCL3L1 copy number, HIV load, and immune reconstitution in sub-Saharan Africans
Source: BMC Infect Dis. 2013 Nov 12;13:536. doi: 10.1186/1471-2334-13-536 (PMC3829100; doi:10.1186/1471-2334-13-536)

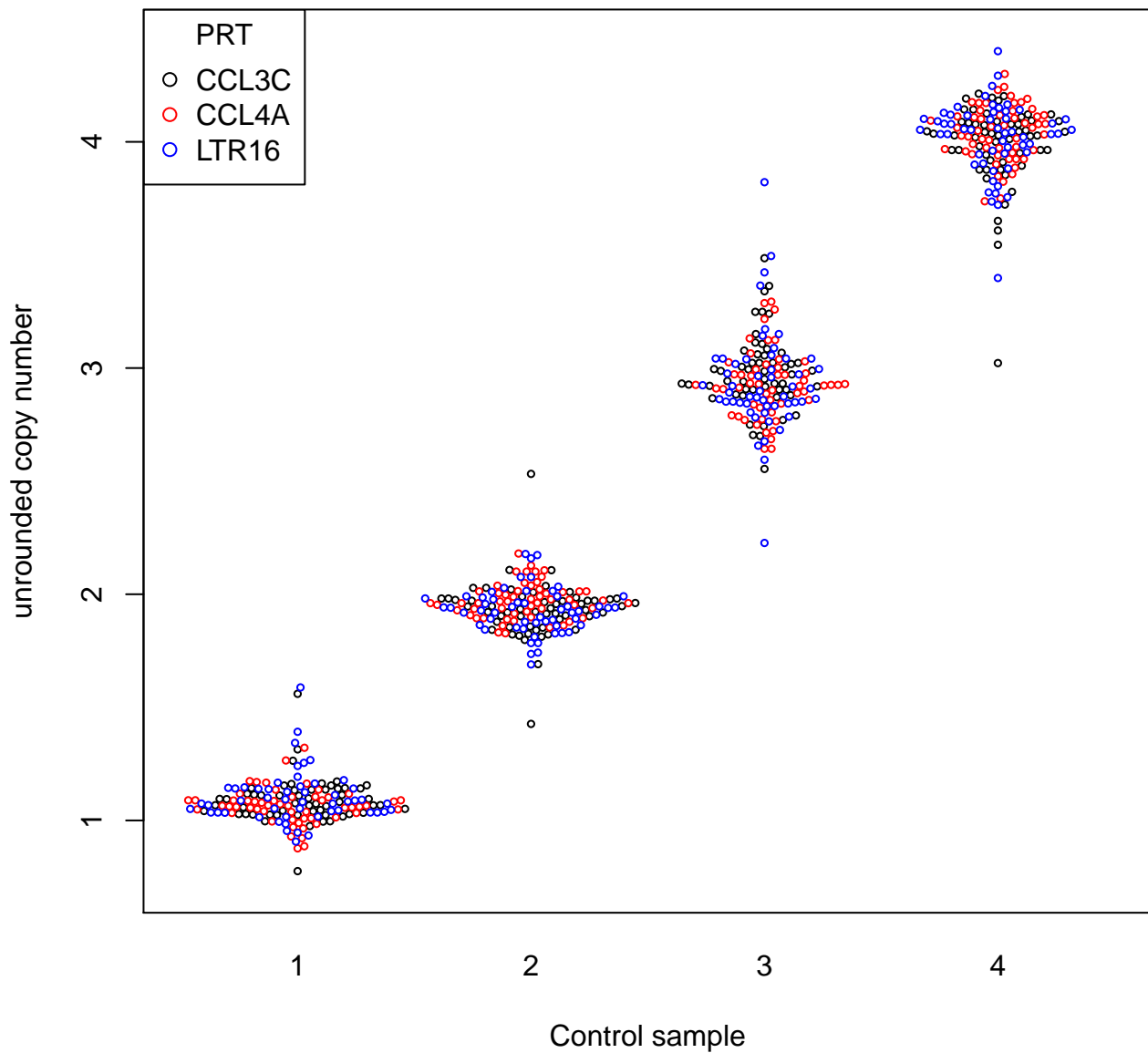

Supplement: Additional file 3: Figure S1 — Analysis of PRT measurement noise in control samples. Individual unrounded PRT values are plotted on the y-axis, according to the different copy numbers of the four controls (x-axis). Each point is coloured according which of the three different PRT assays generated it, all three assays measuring CCL3L1 copy number. [file 1471-2334-13-536-S3.pdf]

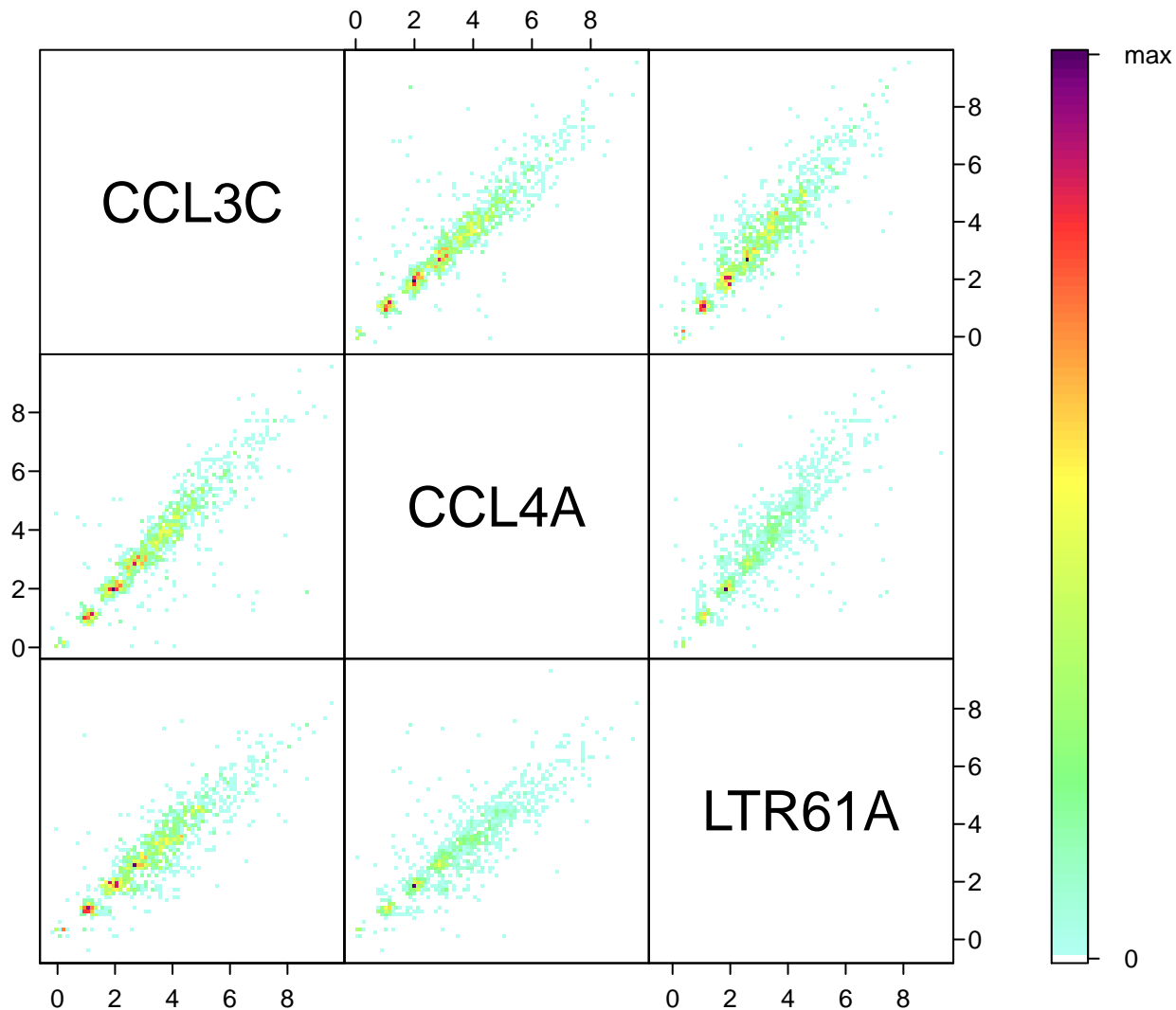

Supplement: Additional file 4: Figure S2 — Clustering of PRT raw data between different assays. For the complete dataset (n = 1133), density scatterplots were draw comparing each of the three different assays with each other. Axis labels indicate raw PRT values, and the colour bar on the left indicates the density of individual datapoints. One extreme point has been omitted. [file 1471-2334-13-536-S4.pdf]

**A**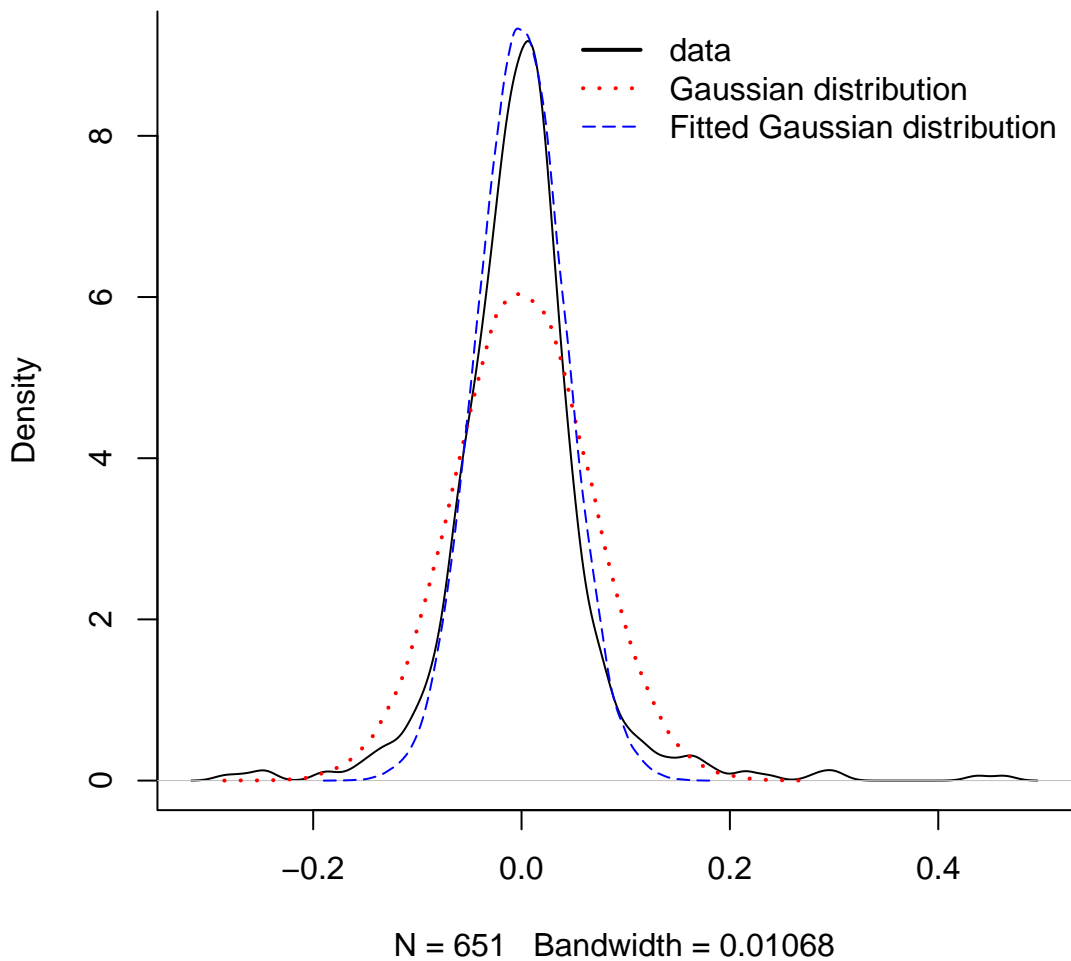**B**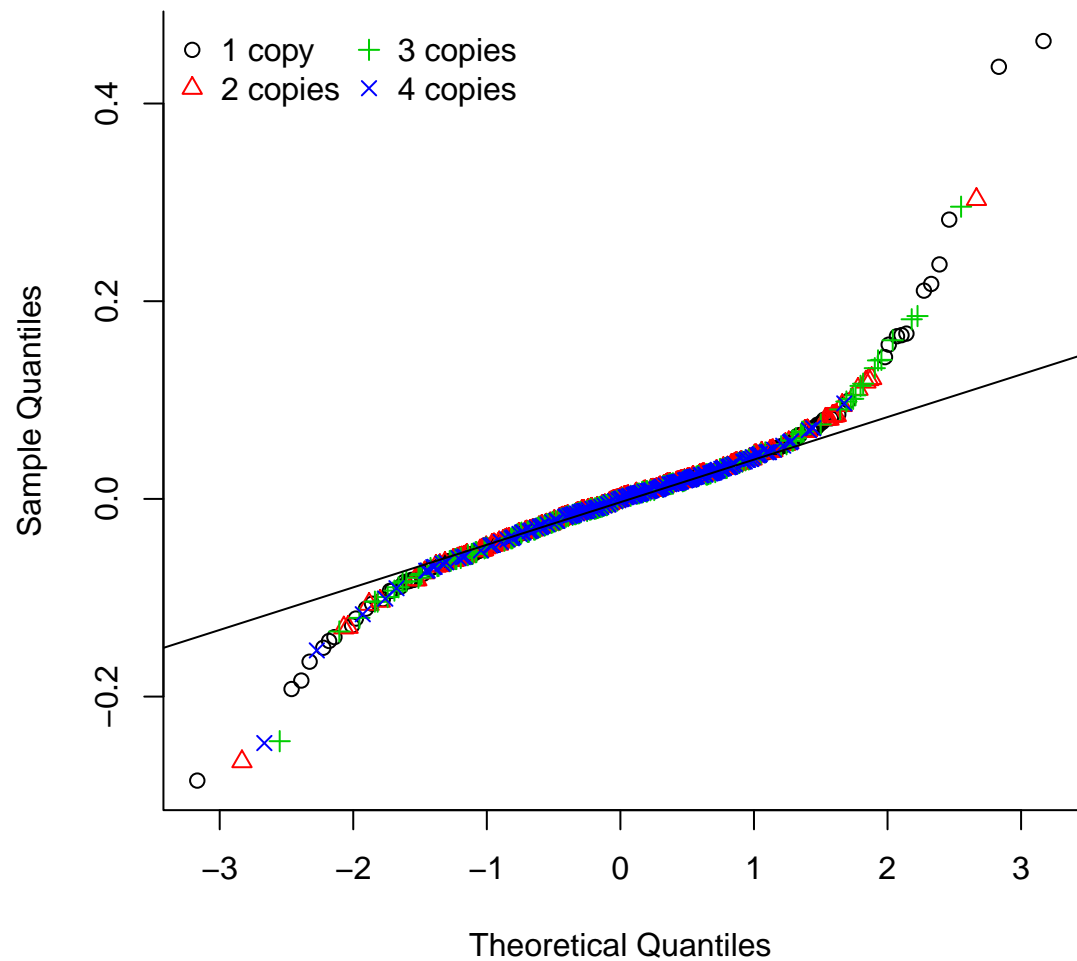

Supplement: Additional file 5: Figure S3 — Analysis of the distribution of PRT values about a single copy number. a). The density of raw unrounded PRT values of the control samples, shown in supplementary Figure 1, is plotted, with values normalised to centre on a mean of zero. The red dotted line represents a Gaussian distribution with a mean and standard deviation taken from the PRT data. The blue dashed line represents a Gaussian distribution fitted to the PRT data. b). Gaussian quantile-quantile plot of raw unrounded PRT values of the control samples. Each value is plotted according the copy number of the control sample, as shown in the legend. The straight line is plotted through the first and third quantiles. [file 1471-2334-13-536-S5.pdf]

Posterior probability of integer copy number call

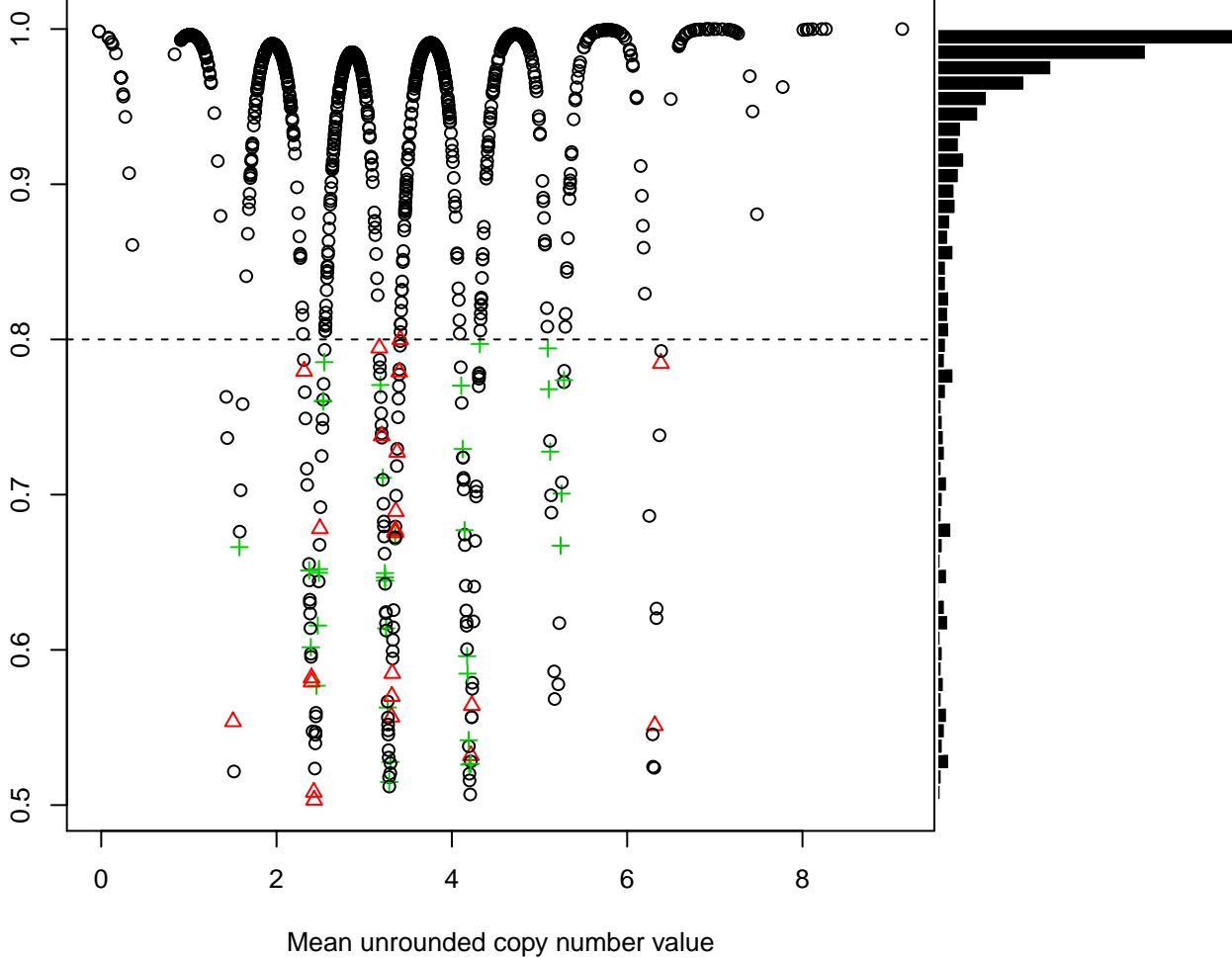

Supplement: Additional file 6: Figure S4 — Confidence of integer copy number calls from raw PRT data.Raw PRT calls of the entire dataset (average of three PRT assays) are plotted on the x-axis with posterior probability of the resulting integer copy number call on the y-axis. Points plotted as red triangles are those where P < 0.8 with a repeat measurement which gave a different estimate of integer copy number (±1). Points plotted as green crosses are those where P < 0.8 with a repeat measurement which gave the same estimate of integer copy number. [file 1471-2334-13-536-S6.pdf]

CCL3C  
CCL4A  
LTR61A

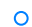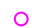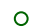

Individual

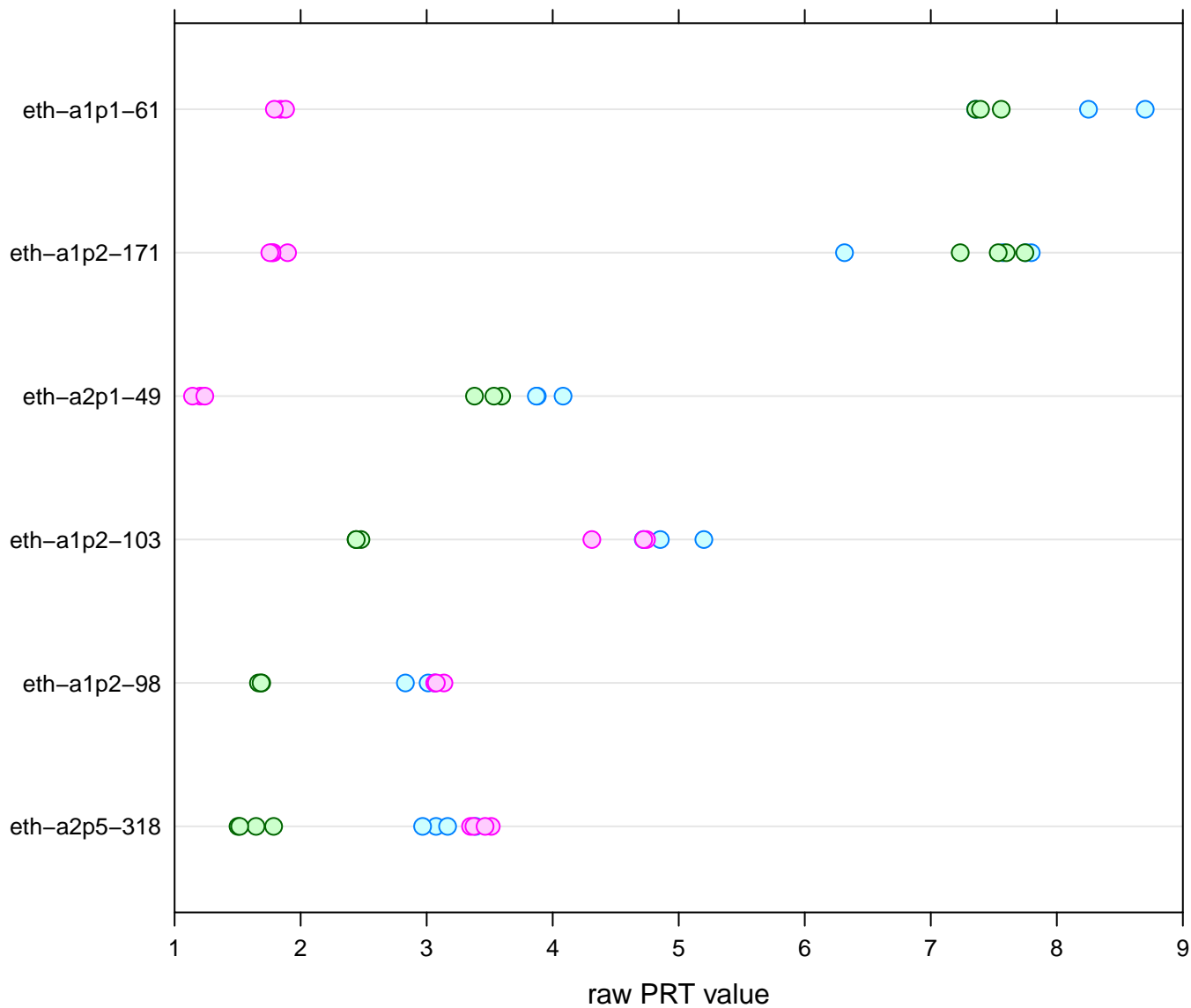

Supplement: Additional file 7: Figure S5 — Examples of assay heterogeneity. Six Ethiopian samples are highlighted, together with the raw PRT ratios, coloured by PRT assay, after several repeat tests. [file 1471-2334-13-536-S7.pdf]
